# Physicochemical behaviour of acetaminophen-Dimethyl Sulphoxide mixtures studied via dielectric spectroscopy

**DOI:** 10.3389/fchem.2026.1733069

**Published:** 2026-05-29

**Authors:** Vinita Khatri, Prasanjit K. Dey

**Affiliations:** 1 Department of Basic Science & Humanities, Mukesh Patel School of Technology Management and Engineering, SVKM’s Narsee Monjee Institute of Management Studies (NMIMS) Deemed-to-be-University, Mumbai, India; 2 Department of Metallurgical Engineering and Materials Science, IIT, Bombay, Mumbai, India

**Keywords:** acetaminophen, dielectric properties, dielectric spectroscopy, Dimethyl sulfoxide (DMSO), drug, cole-cole, conductivity

## Abstract

**Introduction:**

This research examines the dielectric and electrical properties of binary mixtures of acetaminophen (APAP) with Dimethyl Sulfoxide (DMSO) as a means to comprehend their molecular interactions, polarization behaviour, and charge transport mechanisms that are characteristic of pharmaceutical systems.

**Methods:**

The binary mixtures were examined with the help of a broadband dielectric spectrometer, specifically using the Novocontrol Technologies Alpha-A Analyzer, within a frequency range from 10 Hz to 10 MHz at a temperature of 25 °C and the application of a bias voltage of 0.1 V. The variations of the real and imaginary parts of complex permittivity, complex conductivity, and electric modulus with frequency for different APAP concentrations in DMSO were thoroughly studied. Besides, impedance spectroscopy and equivalent circuit modelling were also used to study the interfacial polarization and conduction processes.

**Results:**

Strong electrode polarization effects were found in the dielectric spectra at the low-frequency range, along with evident conduction relaxation behaviour. The concentration-related changes in dielectric parameters revealed strong intermolecular interactions and alterations in charge transport dynamics within the binary mixtures. Impedance analysis and equivalent circuit modelling also supported the existence of interfacial polarization phenomena and generated details about the conductive pathways functioning in the system.

**Discussion:**

This paper unveils the dielectric response and electrical interaction behaviour of the APAP-DMSO mixtures in detail. The results may help future studies seeking to improve pharmaceutical preparations and deepen the understanding of solvent-facilitated molecular interactions in drug delivery systems.

## Highlights

- Objective

The study evaluates the bioavailability of acetaminophen (APAP) in Dimethyl Sulphoxide (DMSO) using dielectric spectroscopy in the frequency range of 10 Hz to 10 MHz.

- Significance

Introduces dielectric spectroscopy as a non-invasive, rapid analytical technique for probing drug-solvent interactions and assessing molecular dynamics.

- Novelty

This is the comprehensive dielectric spectroscopy study of APAP-DMSO binary solutions in the low-frequency range where ionic and electrode polarization dominate.

- Key Findings
  - Dielectric constant (ε′) decreases with frequency; higher values at low frequencies suggest interfacial/space charge polarization.
  - Dielectric loss (ε'') and AC conductivity (σ′) correlate strongly with drug concentration, showing higher values for higher molarity solutions.
  - Relaxation times (τ_EP_ and τ_σ_) show that electrode polarization dominates at low frequency while dipolar relaxation dominates at high frequency.
  - Impedance analysis reveals that higher APAP concentrations exhibit higher interfacial polarization, higher double-layer capacitance, and lower resistance, suggesting enhanced molecular mobility.
- Methodology
  - Five APAP-DMSO solutions were prepared with increasing molarity.
  - Dielectric measurements were performed at 298.15 K using Novocontrol Alpha Analyzer.
  - Impedance spectroscopy data were fitted using equivalent circuit models tailored for each concentration.
- Implications
  - Strong correlation between dielectric/impedance parameters and drug molecular mobility.
  - Highlights the potential of formulation optimization using DMSO to enhance APAP’s solubility and absorption.
  - Establishes a foundation for future model-based studies using dielectric techniques.
- Technical Strength

Utilizes multiple electrical parameters (ε′, ε'', σ′, σ'', Z, M″, tan δ) for comprehensive analysis.

Applies Jonscher’s power law, modulus formalism, and Nyquist plots to decode molecular interactions.

- Conclusion

The study demonstrates that dielectric and impedance measurements can effectively elucidate solute-solvent interactions relevant to formulation strategies.

## 1 Introduction

Dielectric spectroscopy provides one of the most powerful means for studying the molecular assembly and dielectric relaxation processes. Dielectric parameters are very sensitive to interaction strength, degree of solvation, and mobility of dipolar or ionic species in the medium (Kremer and Schönhals, 2002). The size and the changes of dielectric relaxation give direct information about the microscopic surroundings, such as the formation of the hydrogen bonds, dipolar coupling, and local viscosity effects (Jonscher, 1999; Ngai, 2011), i.e., the physicochemical behaviour. Uncontrolled variations in formulation can lead to unpredictable physicochemical behaviour. Therefore, it is very important to understand the dielectric response in order to know the formulation dynamics of the systems (Florence and Attwood, 2015; Maggio et al., 2013). A dielectric profile that is well understood can help to forecast the performance of a system under working conditions. Limited molecular mobility can lead to the loss of functional efficiency, while very high mobility causes instability or degradation (Ioele et al., 2022). Even though dielectric behaviour is only one aspect of the broader physicochemical landscape, it still serves as a vital indicator of molecular organization, interaction strength, and structural dynamics in complicated systems (Debenedetti and Stillinger, 2001). Innovations, such as nanoscale materials, specialised delivery platforms, and micro-scale interfacing methods have emphasized the need for dielectric analysis to probe subtle interaction-driven changes in advanced material systems (Baker et al., 2014; Mogbojuri et al., 2025).

This paper focuses on a widely used analgesic acetaminophen (N-acetyl p-aminophenol, APAP), employing Dielectric Relaxation Spectroscopy (DRS) to investigate its properties (Cheng et al., 2013; Ohashi and Kohno, 2020; van Rensburg and Reuter, 2019). APAP does not produce gastrointestinal harm or troublesome cardiorenal effects as NSAIDs does (Alchin et al., 2022; Russell et al., 2003). It works on the parts of brain that receive the ‘pain messages.’ Pharmacy, drug management, medicine, these are few among the areas where dielectric studies play a vital role. Dielectric properties facilitate in optimizing the granulation process for different oral dosage forms. Dielectric relaxation spectroscopy (DRS) plays a vital role in exploring the electric polarization process by measuring the polarizability of a material in a weak electromagnetic field. This technique offers a rapid and non-invasive approach for probing the structural and physicochemical properties of pharmaceutical materials. DRS acts as a source to study molecular interactions and thus determining molecular dynamics. This technique can be implemented for a wide frequency, ranging from 10^–6^–10^12^ Hz and thus covers wide applications in different sectors including pharmaceutics (Hiremath et al., 2015; Kremer et al., 2022; Raicu and Feldman, 2015; Rana and Pandit, 2019). Literature (Mahdi et al., 2020) shows that APAP with Dimethyl Sulphoxide (DMSO) is untouched in 10^–6^–10^12^ Hz frequency range. Considering this fact, we have chosen low frequency region to study dielectrics of APAP with DMSO. Electrode polarization and ionic polarization effects are predominant compared to dipolar relaxation in this range. The optimum frequency range can be determined by studying the dielectric properties with frequency. The relation between frequency and dielectric properties directs to which application can be focused on for the chosen material (Dey and Bhattacharyya, 2026; Wang et al., 2001).

Dimethyl sulfoxide (DMSO), a polar aprotic solvent, is widely used in chemical and biomedical applications due to its high dielectric constant and amphiphilic nature, enabling it to solubilize a broad array of organic compounds. It is a molecule with a long history in pharmaceutics. It is also used in extraction and coating processes due to its capacity to solubilize a broad spectrum of compounds, including both polar and nonpolar substances. A group of researchers suggested that both unintentional and intentional adverse effects associated with APAP could potentially be mitigated by replacing conventional APAP with an APAP-DMSO formulation (Algfeley et al., 2019). Concurrently, the definitive pharmacodynamic mechanism of APAP within the human body has yet to be fully elucidated (Bessems et al., 2001). To address this gap, the dielectric and electrical characteristics of APAP–DMSO mixtures were examined in the frequency window of 10 Hz–10 MHz at 25 °C. The investigation includes measuring complex permittivity, conductivity, electric modulus, and impedance spectra across varying APAP concentrations. By coupling experimental data with impedance modelling and equivalent circuit analysis, we identify key relaxation phenomena and their dependence on concentration, highlighting the effects of molecular interactions on charge dynamics. The measured dielectric properties are indicative of the changes in molecular interactions and the solvation behaviour which can have an effect on the properties of the formulation. However, their implication on the formulation characteristics should be confirmed by further pharmaceutical studies. This study uniquely presents a systematic concentration-dependent dielectric investigation of APAP–DMSO mixtures, elucidating molarity-driven changes in polarization and relaxation behaviour.

## 2 Experimental

### 2.1 Materials and measurements

Acetaminophen powder obtained from Farmsons Pharmaceuticals Gujarat Pvt. Ltd., Vadodara, India is 99.9% pure and Dimethyl Sulphoxide (DMSO) purchased from Chemtek Scientific Private limited of India is >99.9% pure. The solubility of APAP in DMSO is 20mg/mL as mentioned in the product information by Cayman Chemical Company. The company delivers chemicals predominantly used in academic and pharmaceutical research, particularly for drug development. Therefore, we prepared total five solutions in steps of 100 mg of APAP in different volume of DMSO maintaining the mentioned solubility. The APAP powder is dissolved in the solvent (DMSO) with the help of magnetic stirrer and thus used for further analysis. The final solution volumes were measured after complete dissolution using a graduated measuring cylinder, with an estimated measurement uncertainty of ±0.1 mL, prior to analysis. Considering the typical working volume (e.g., 10–20 mL), the estimated uncertainty in concentration is within ∼0.5–1%. Care was taken to minimize systematic errors by maintaining consistent preparation conditions for all samples. This level of uncertainty is small compared to the overall variation observed in the measured dielectric parameters and does not affect the observed trends. The molarity of all the solutions were found using Equations 1, 2.

$$M=\frac{n}{V}$$ (1)

where, M is the molarity (mol L^−1^), n is the amount of APAP in moles (mol), and V is the total volume of the solution (L).

$$\text{here},n=\frac{m}{\text{Mr}}$$ (2)

where m is the mass of APAP (g) and Mr​ is the molar (relative molecular) mass of APAP (g mol^−1^).

Dielectric and electric parameters of the binary mixtures of APAP with DMSO were determined at 25 °C, by using Novocontrol Alpha analyser in an alternating current frequency range from 10 Hz to 10 MHz. Calibration of the instrument is done first with the standard liquid and then the samples were analysed using the test fixture for liquid samples. The measurements were performed with limited replicates (n = 2) under identical experimental conditions. The variation between replicate measurements was within ± 1%–2%, consistent with instrumental precision and therefore statistical variability cannot be fully evaluated.

## 3 Data analysis and interpretation

### 3.1 Dielectric and electric properties

Ionic conduction and electrode polarization are observed at frequencies below 0.1 MHz, which is clearly the resultant of the increase in dielectric constant values in that range (Reis et al., 2009; Sengwa and Sankhla, 2007). The dielectric constant is a parameter which varies with refractive index, viscosity and dissolution rate etc (Sengwa et al., 2011). Solvents with higher dielectric constants typically exhibit greater solute-dissolving capability (Jouyban et al., 2004).

**FIGURE 1:**
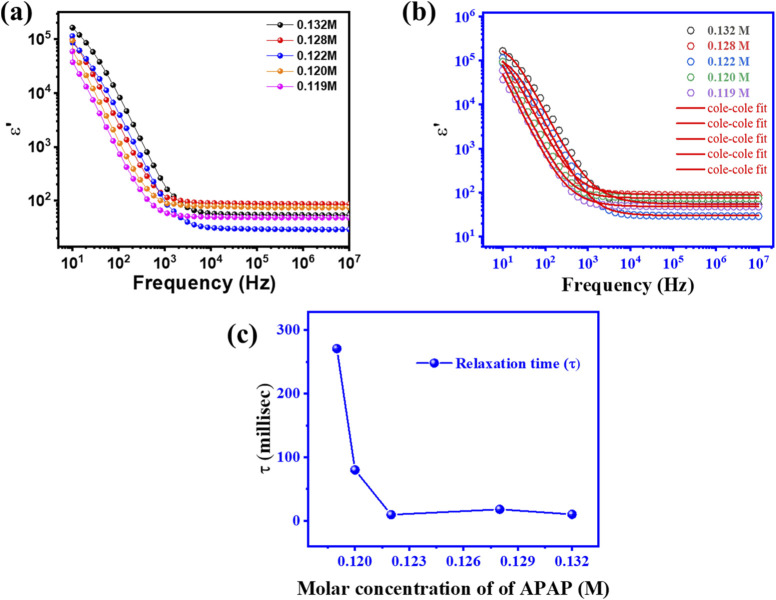
**(a)** Dielectric constant (ε′) spectra for various concentrations of APAP in DMSO at 25 °C temperature for frequency varying between 10 Hz and 10 MHz, **(b)** Dielectric constant (ε′) spectra for various concentrations along with Cole-Cole fit of APAP in DMSO at 25 °C temperature for frequency varying between 10 Hz and 10 MHz, and **(c)** Relaxation time as a function molar concentration of APAP.

Figure 1a show the dielectric constant (ε′) for different molarity solutions of APAP for the full frequency range. The ε′ values decreased with the reduction in molar concentration of solution. At lower frequencies the elevated ε′ values suggest the presence of Maxwell-Wagner-Sillars polarization, leading to the modification of the field distribution (Kyritsis et al., 1995; Yue et al., 2022). With the increase in frequency the accumulation of ions of conducting materials stops due to the lack of time. This results into frequency-independent values of ε′ at higher frequencies (more than 1 kHz) for all molar solutions of APAP. In the high-frequency region, where mobile charge carriers are unable to follow the rapid field oscillations, conduction, and Maxwell–Wagner polarization effects diminish significantly (Böttger and Bryksin, 2022; Sardar et al., 2025). Beyond 1 kHz, ε′ remains nearly constant, which shows the rigidity of the molecules with changing applied field. A higher ε′ is indicative of an increasingly polar medium and stronger solute–solvent interactions, which are qualitatively consistent with enhanced solvation behaviour reported in the literature (Sahoo et al., 2025; Zhang et al., 2012). The Cole-Cole fitting was done to obtain the relaxation parameters of the ε′ spectra and is shown in Figure 1b. The Cole-Cole model equation is given below as Equation 3,

$$\varepsilon *=\varepsilon_{\infty }+\frac{\varepsilon_{s}-\varepsilon_{\infty }}{1+{\left(j\omega \tau \right)}^{\alpha }}$$ (3)

where, $\varepsilon_{s}$ and $\varepsilon_{\infty }$ represent the static (dc) and high-frequency permittivity, respectively. The broadening factor (α) controls the distribution of an enormous number of molecular relaxations, seen in the ε′ at the Debye peak or critical relaxation frequency, fc = (2πτ) ^−1^ and 0 ≤ α ≤ 1. The fitted parameters are tabulated in Table 1. Increase in α indicates a broader distribution of relaxation times and deviation from single-time-constant behaviour. Figure 1c showing the decrease in relaxation time with the increase in molar concentration which implies faster dipolar reorientation and enhances the system’s ability to polarize under an applied field. This behaviour is consistent with the ε′ spectra, where more rapid relaxation tends to maintain higher permittivity at low frequencies and shifts the dielectric dispersion toward higher frequencies.

**TABLE 1:** Cole-cole fitting parameters of real permittivity (ε′) for various molar concentrations of APAP.

Molar concentration (M)	* $\varepsilon_{\infty }$	(Δ ε ± error) x 10^5^	(τ (sec) ± error) x 10^–2^	*Alpha (α)	R^2^ (COD)
0.132	55	(2.28 ± 0.00)	(1.01 ± 0.007)	0.014	0.998
0.128	90	(2.17 ± 0.04)	(1.79 ± 0.025)	0.012	0.999
0.122	30	(1.26 ± 0.03)	(0.95 ± 0.000)	0.007	0.969
0.120	75	(19.92 ± 0.59)	(8.00 ± 0.000)	0.002	0.961
0.119	48	(136.73 ± 0.92)	(94.20 ± 0.003)	0.001	0.959

* The values of $\varepsilon_{\infty }$ and α are exact; no uncertainty is associated.

**FIGURE 2:**
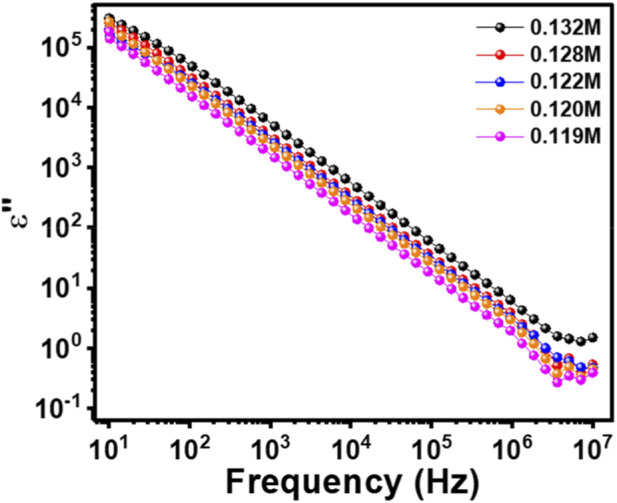
Dielectric loss (ε′′) spectra for various concentrations of APAP in DMSO at 25 °C temperature for frequency varying between 10 Hz and 10 MHz.

In Figure 2, the deviation in the dielectric loss (ε′′) value is seen from 20 Hz–150 Hz at constant temperature 25 °C. All the samples show exponential decrease, the sample with the highest molarity has the highest loss, and the loss decreases as the molarity decreases through the entire investigated frequency range. ε′′ indicates the amount of heat generated per unit time when subjected to microwave heating. Overheating and under heating causes damage to drugs (Khatri and Dey, 2025; Magee et al., 2013; Stahl, 2025; Tang et al., 2018). On comparing Figures 1, 2 it is observed that for the lower frequencies from 10 Hz to near around 25,000 Hz, the ε′′ is dominant over the polarization of the solution. At low frequencies, electrode polarization dominates the dielectric response, whereas at higher frequencies, dipolar relaxation and conduction processes are the primary contributors.

The molecular reorientation dynamics are seen to be changed by the dispersion observed. Cole–Cole plots exhibit depressed semicircular arcs, indicating deviation from ideal Debye relaxation. Non-zero values of the Cole–Cole distribution parameter (α) suggest a distribution of relaxation times, consistent with the formation of heterogeneous microenvironments arising from solute–solvent interactions.

**FIGURE 3:**
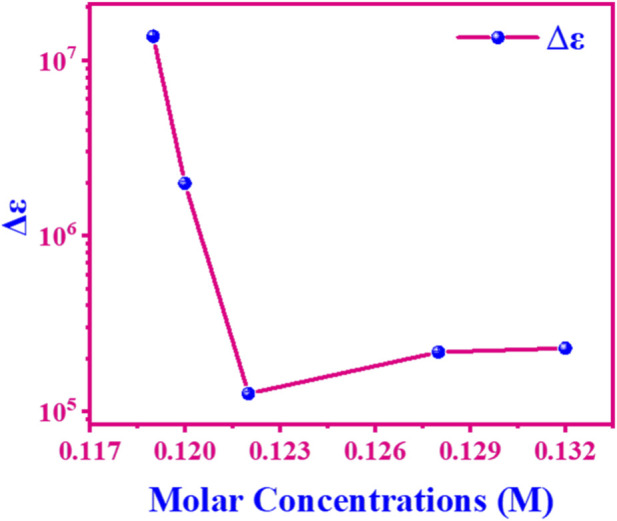
Variation of relaxation strength (Δε) with molar concentrations of APAP in DMSO at 25 °C.

The dielectric strength (Δε) exhibits a highly non-linear variation with molar concentration as seen in Figure 3. While Δε remains relatively stable (∼10^5^) at higher concentrations (0.122–0.132 M), a pronounced increase to the order of 10^6^–10^7^ is observed near 0.120–0.119 M. This anomalous enhancement indicates the onset of strong intermolecular interactions and cooperative dipolar dynamics, possibly arising from aggregation or structural reorganization in the system.

**FIGURE 4:**
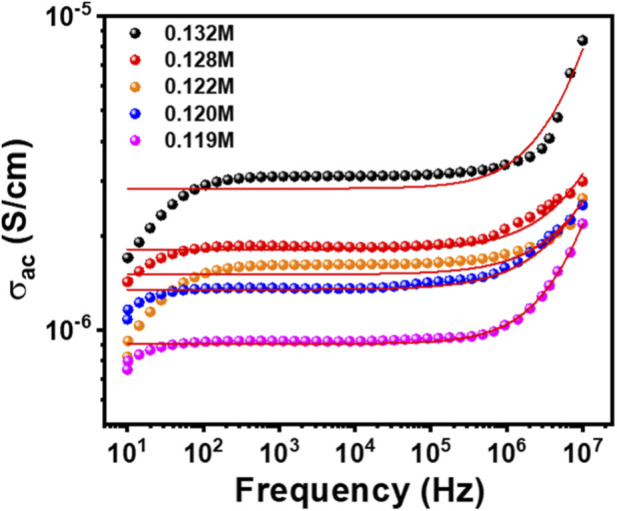
Frequency dependent real part σ′ of ac complex conductivity for different concentrations of APAP in DMSO at 25 °C.

The spectra of σ′ (real part of alternating current (ac) complex conductivity) as a function of frequency for all the samples of APAP in DMSO at 25 °C is portrayed in Figure 4. As observed, ac conductivity of all samples is higher in frequency region (10^6^–10^7^ Hz) which is just the opposite in case of ε′ spectra. While the change in ε′ is because the added molecules have different polarization than the host molecules. It has been observed that the solution with maximum molarity has the highest value of ac conductivity which keep on decreasing with the decrease in the molarity of APAP. Negligible change in ac conductivity values is observed in the frequency range 100 Hz till 10 MHz which is followed by drastic increase.

The apparent rise in σ′ at high frequencies is consistent with the behaviour observed in the ε′ and ε″ spectra. As shown in Figures 1, 2, both ε′ and ε″ gradually level off toward the upper end of the measured frequency range, confirming that electrode-polarization and dc-conduction effects become negligible in this region. This high-frequency plateau does not indicate a dipolar relaxation process; rather, it reflects the intrinsic, frequency-independent dielectric response of the material once interfacial and space-charge contributions have diminished.

In Figure 4, the increase in σ′ observed in the high-frequency region (10^6^–10^7^ Hz) corresponds to a subtle change also visible in the ε″ spectrum, where a slight upward deviation appears above 1 MHz. This feature does not signify the onset of a dipolar relaxation, as no characteristic signatures such as a step in ε′ or a peak in ε″ are present within the measured range. Instead, the high-frequency response reflects the diminishing influence of electrode polarization and conduction effects, revealing the intrinsic, nearly frequency-independent dielectric behaviour of the material. The mild upturn in ε″ and the accompanying increase in σ′ therefore arise from the same underlying high-frequency polarization processes and not from a relaxation mechanism. Overall, the ε′, ε″, and σ′ spectra are mutually consistent.

The value of σ′ is highest for 0.132 M solution which keeps on reducing with the decrease in molar concentration in the measured frequency range. In the frequency range 500 Hz - 1 MHz, the σ′ spectra exhibit a plateau indicative of the direct current (dc) conductivity (σ_dc_), whereas the frequency range 10 Hz–500 Hz shows a pronounced decline attributable to electrode polarization (EP) effects. The σ_dc_ values are determined by doing the Jonscher power law fitting Equation 4,

$$\sigma_{ac}=\sigma_{dc}+A\omega^{n}$$ (4)

where, σ_ac_​ is the total conductivity, σ_dc_​ represents the frequency-independent dc conductivity corresponding to long-range charge transport, A is a temperature-dependent pre-exponential factor, ω is the angular frequency (ω = 2πf), and n is the frequency exponent (0 < n < 1) that reflects the nature of charge transport and degree of interaction among charge carriers. The σ_dc_ values are reported in Table 2, which is decreasing with the increase in the molarity of APAP in the solutions.

**TABLE 2:** Joncher’s power law fitting parameters for various molar concentrations of APAP.

Molar concentration (M)	σ_dc_ (S/cm) x 10^–6^	A x 10^–13^	n	R^2^(COD)
0.132	0.90	0.41	0.96	0.995
0.128	1.35	7.20	0.80	0.996
0.122	1.51	4.29	0.82	0.997
0.120	1.81	3.78	0.84	0.997
0.119	2.83	1.14	0.98	0.995

To avoid ambiguity between different polarization mechanisms, the dielectric spectra have been re-analyzed by separately identifying the contributions of electrode polarization (EP), interfacial (Maxwell–Wagner–Sillars) polarization, and conduction relaxation. The low-frequency divergence of ε′ and the steep increase in ε″ are now attributed explicitly to EP, which arises from space-charge accumulation at the blocking electrodes. The intermediate-frequency dispersion is described in terms of interfacial polarization originating from heterogeneities in the sample. In contrast, the conduction relaxation process is isolated from the bulk response and quantified using model-based fitting (Cole–Cole), rather than relying solely on the positions of graphical peaks. This revised analysis ensures that each mechanism is interpreted independently and prevents overlap among EP, conduction relaxation, and interfacial effects.

**FIGURE 5:**
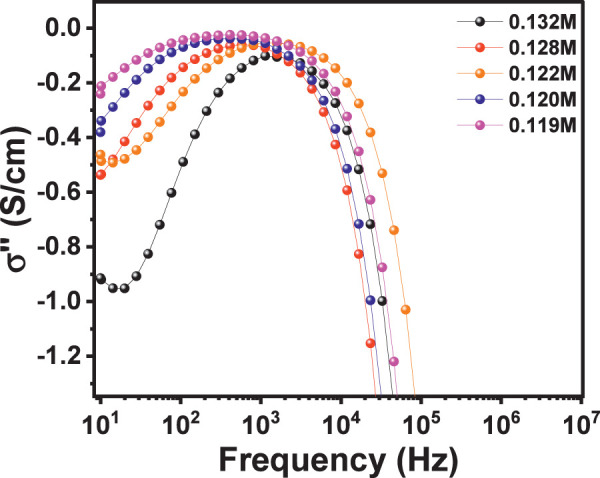
Variation of σ″ (imaginary component of ac complex conductivity) with frequency 10 Hz–10 MHz for various molar concentrations of APAP in DMSO at 25 °C.

**FIGURE 6:**
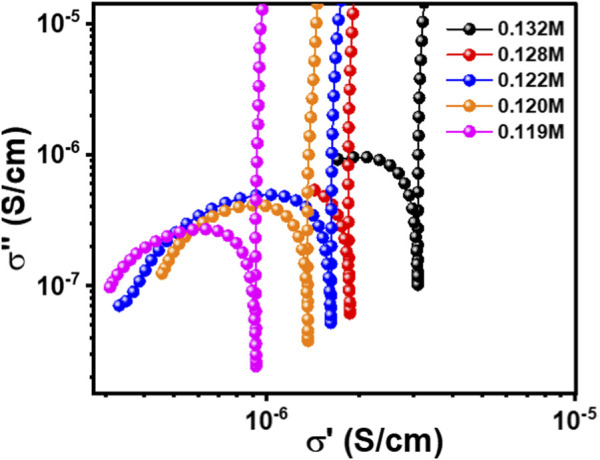
σ″– σ′ complex plane representation of ac conductivity for various molar solutions of APAP in DMSO at 25 °C.

Figure 5 depicts the spectral response as a function of frequency of σ″ (imaginary part of ac complex conductivity) for APAP–DMSO mixtures at 25 °C, revealing a relaxation response appearing in the EP-governed region (20 Hz–10 kHz) across all samples. It is observed that at higher frequencies i.e., (10 kHz-10 MHz) values of σ″ for all samples dropped exponentially. Figure 6 shows complex conductivity plots (σ″ vs. σ′) for APAP–DMSO mixtures at 25 °C. All samples exhibit a well-defined semicircle in the low-frequency region and a vertical spike in the high-frequency region, indicative of ionic transport processes at low frequencies and dipolar relaxation mechanisms at the onset of high frequencies.

**FIGURE 7:**
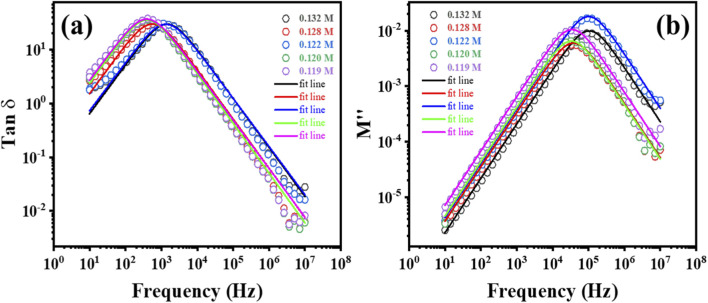
**(a)** Tangent loss spectra (tan δ) **(b)** Imaginary part (M″) of complex electric modulus for all molar solutions of APAP-DMSO at 25 °C for frequency varying between 10 Hz and 10 MHz.

Figures 7a,b portrays the representation of frequency dependent dielectric loss (tan δ) and electric modulus loss (M″) characteristics for all concentration solutions at 25 °C, respectively. The spectra reveal that M″ values increase linearly in the low-frequency region (10 Hz–1000 Hz) for all molar solutions, followed by distinct relaxation peaks at higher frequencies. Each peak represents a conduction relaxation process at its characteristic frequency (f_σ_), from which the conduction relaxation time (τ_σ_) can be obtained using Equation 5; (Sengwa et al., 2017);

$$\tau_{\sigma }=\left(2\pi f_{\sigma }\right){\;}^{-1}$$ (5)

But the peaks in the dielectric loss tangent (tan δ) spectrum are observed at the intermediate frequency range of 500 Hz–1,000 Hz. This feature is associated with the electrode polarization frequency (f_EP_), from the EP relaxation time (τ_EP_) using Equation 6 can be obtained.

$$\tau_{\text{EP}}=\left(2\pi f_{\text{EP}}\right){\;}^{-1}$$ (6)

Values of *τ*_EP_ and *τ*_σ_ can be calculated using Equations 5, 6, however here we have reported the relaxation time obtained from the curve fitting rather than from the peak position directly as shown in Figures 7a,b, and reported in Tables 3, 4. It has been observed that electrode polarization relaxation time is almost 100 times high as compared to conduction relaxation time, which indicates that the molecules of all the samples regain the disorder in the higher frequency range more quickly as compared to low frequency values.

**TABLE 3:** Electrode polarization relaxation time (τ_EP_) obtained from fitting dielectric loss tangent (tan δ) for various molar concentrations of APAP.

Molar concentration (M)	A ± error	τ_EP_ (sec) ± error x 10^–6^	Alpha (α) ± error	R^2^(COD)
0.132	59.50 ± 0.55	(110.0 ± 1.00)	0.91 ± 0.010	0.996
0.128	59.72 ± 0.62	(288.0 ± 4.42)	0.91 ± 0.012	0.995
0.122	59.40 ± 0.56	(118.2 ± 1.65)	0.89 ± 0.010	0.996
0.120	71.69 ± 0.53	(402.5 ± 4.27)	0.92 ± 0.009	0.997
0.119	74.10 ± 0.56	(408.0 ± 4.56)	0.90 ± 0.008	0.998

**TABLE 4:** Conduction relaxation time (τ_σ_) obtained from fitting M‘‘ for various molar concentrations of APAP.

Molar concentration (M)	$M_{\infty }$	(τ_σ_ (sec) ± error) x 10^–6^	Beta (β)	R^2^(COD)
0.132	0.020	(1.52 ± 0.04)	0.98	0.986
0.128	0.012	(4.16 ± 0.07)	0.98	0.992
0.122	0.036	(1.57 ± 0.03)	0.98	0.993
0.120	0.013	(4.74 ± 0.02)	0.98	0.999
0.119	0.021	(4.57 ± 0.04)	0.98	0.998

* The values of $M_{\infty }$ and β are exact; no uncertainty is associated.

Dielectric properties of APAP-DMSO mixtures are a reflection of how intermolecular interactions influence the processes of polarization and relaxation. The composition of APAP, which includes phenolic and amide groups, allows it to interact through hydrogen bonding with the solvent, but the strongly aromatic structure of the molecule constrains the interactions mainly to localized ones rather than the formation of networks of dipoles. This is why the presence of solute changes the local environment of solvent dipoles and limits their rotational mobility, with the consequence that the dielectric response varies with the change in frequency.

### 3.2 Impedance analysis

**FIGURE 8:**
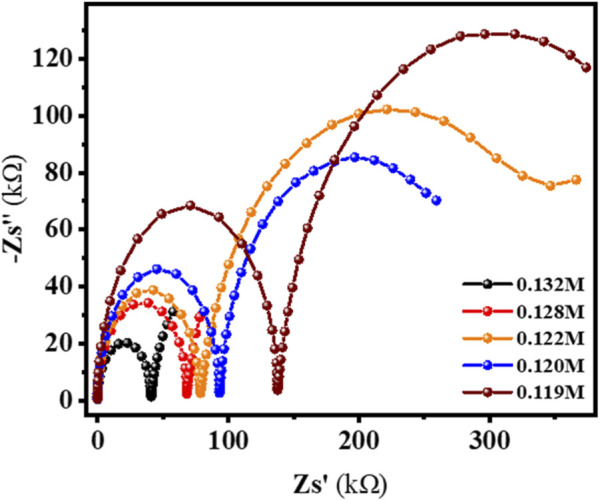
Impedance spectra in the complex plane (−Zs″ vs. Zs′) for all APAP–DMSO binary solutions at 25 °C across the frequency range 10 Hz–10 MHz.

Impedance spectroscopy was performed on various molar concentrations of APAP in DMSO at 25 °C, within the investigated frequency range 10 Hz to 10 MHz. Nyquist plots (Figure 8) were used to represent the impedance spectra, providing insight into the electrical properties and interfacial processes of the samples.

**FIGURE 9:**
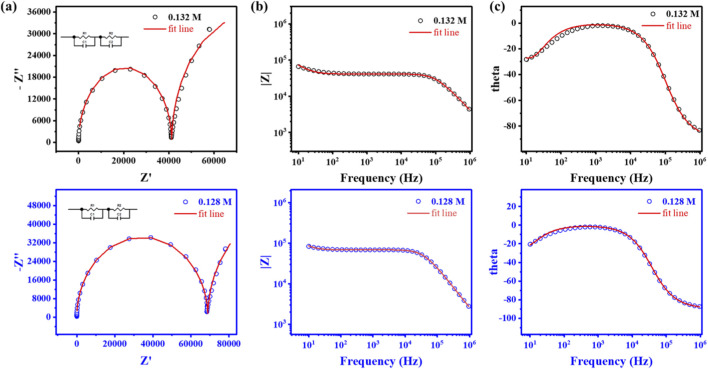
Impedance spectra of APAP–DMSO solutions (0.132 M and 0.128 M) at 25 °C: **(a)** Nyquist plot (−Zs″ vs. Zs’), Bode plot shown in **(b)** |Z| vs. frequency, and **(c)** θ vs. frequency.

The Nyquist plots for the initial two samples exhibited well-defined semicircular arcs, which were accurately modelled using an equivalent circuit comprising two parallel Resistor–Capacitor (R-C) circuit model. This model represents distinct bulk and interfacial relaxation processes within the system. As shown in the Nyquist and Bode plots (Figure 9), the fitting demonstrates an excellent correlation between experimental observations and simulated data.

**FIGURE 10:**
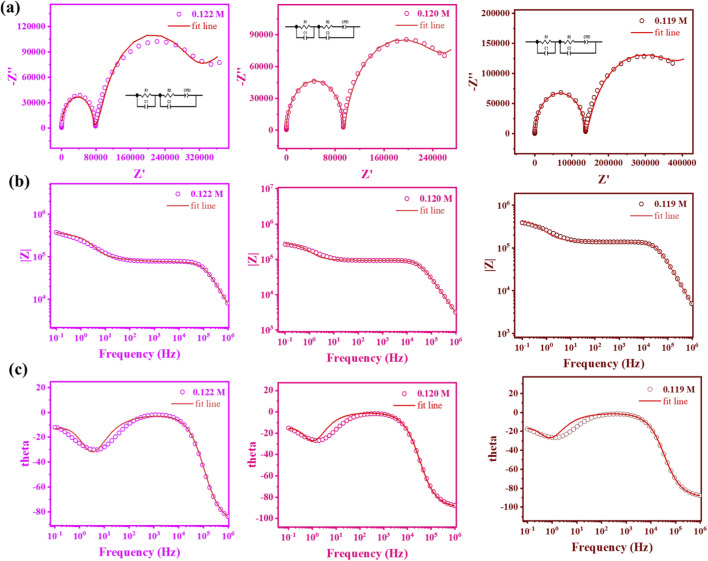
Impedance spectra of APAP–DMSO solutions (0.122 M, 0.120 M, 0.119 M) at 25 °C: **(a)** Nyquist plot (−Zs″ vs. Zs’), Bode plot shown in **(b)** |Z| vs. frequency, and **(c)** Theta vs. frequency.

For the remaining three samples, the Nyquist plots displayed a characteristic “tail” at the low-frequency end of the semicircle, indicating a more complex interfacial phenomenon. These samples are best represented by a modified equivalent circuit comprising a single R–C branch in series with a parallel combination of a resistor (R) and a constant phase element (CPE1), both connected in parallel with a capacitor (C), as illustrated in Figure 10. This configuration accounts for the additional dispersion and frequency-dependent behaviour arising from inhomogeneities or diffusion-related processes in the system.

The fitting parameters obtained from the equivalent circuit models are summarized in Table 5. These include charge transfer resistances (Rct_1_, Rct_2_), double-layer capacitances (Cdl_1_, Cdl_2_), and constant phase element parameters (CPE-T, CPE-P), representing interfacial charge transfer processes and non-ideal capacitive behaviour arising from surface heterogeneity. A clear trend of increasing Rct and Cdl with rising APAP concentration was observed across all samples. The observed increase in Rct signifies hindered interfacial charge transport, leading to a corresponding decrease in charge carrier mobility, likely due to enhanced molecular interactions or aggregation at higher drug concentrations, which impedes ionic transport. Simultaneously, the significant enhancement in Cdl reflects stronger interfacial charge accumulation at reduced concentrations, indicating that a larger number of charge carriers are being stored at the interface rather than being transported across it. Such an increase in interfacial capacitance can be attributed to slower charge-transfer kinetics across the interface. As the molar concentration decreases, the availability and mobility of charge carriers are reduced, leading to delayed interfacial exchange. Consequently, charges tend to accumulate near the interface, giving rise to an elevated double-layer capacitance. In addition, the enhanced Cdl suggests increased charge trapping at interfacial states and a higher degree of electrode polarization. The accumulation of trapped charges creates localized electric fields that further impede charge transport, reinforcing polarization effects. The simultaneous increase in both Rct and Cdl therefore indicates a scenario in which charge carriers are effectively confined at the interface but are unable to participate efficiently in long-range transport. This behaviour is characteristic of mobility-limited charge transport, where interfacial barriers dominate over bulk conduction pathways. The Bode plots further corroborate these findings by showing frequency-dependent variations in impedance magnitude and phase angle consistent with the fitted equivalent circuits, confirming the validity of the models used.

**TABLE 5:** Equivalent circuit model fit parameters for various molar concentrations of APAP.

Molar concentration (M)	Rct1 × 10^5^	Error (Rct1)	Cdl1	Error (Cdl1)	Rct2 × 10^5^	Error (Rct2)	Cdl2	Error (Cdl2)	CPE1-T	CPE1-P
0.132	0.41	240.32	3.83 × 10^−13^	4.007 × 10^−13^	0.7292	3782.0	3.49 × 10^−7^	9.352 × 10^−9^	—	—
0.128	0.69	188.76	6.19 × 10^−11^	2.445 × 10^−13^	0.9818	6108.1	4.53 × 10^−7^	8.527 × 10^−9^	—	—
0.122	1.65	216.40	5.17 × 10^−7^	1.145 × 10^−9^	0.7350	1,056.5	2.34 × 10^−11^	2.534 × 10^−13^	8.13 × 10^−6^	0.349
0.120	1.29	324.60	1.75 × 10^−6^	3.215 × 10^−8^	0.9373	3244.8	5.22 × 10^−11^	6.839 × 10^−13^	1.69 × 10^−5^	0.549
0.119	1.75	288.20	1.69 × 10^−6^	2.614 × 10^−8^	1.3632	2765.2	3.37 × 10^−11^	3.664 × 10^−13^	9.26 × 10^−6^	0.480

Overall, the impedance analysis elucidates the impact of molar concentration on the electrical and interfacial properties of the APAP-DMSO system, complementing the dielectric spectroscopy results and offering valuable insights into the molecular interactions influencing relaxation dynamics. At very low frequencies, the polarization of electrodes and interfaces is dominating, whereas at the highest frequencies, the dipolar relaxation determines the behaviour. The Cole-Cole plots reveal that relaxation cannot be described by the Debye model, which means there is a range of relaxation times due to different solute-solvent interactions providing some heterogeneity to the microenvironments. To sum up, these points highlight that localized hydrogen bonding and constrained motion of molecules are important factors affecting the dielectric response of the system.

### 3.3 Limitations of the work

The current study has a limitation in that all the measurements were taken only at 25 °C and within a limited concentration range. Although conditions of this kind may serve as a controlled basis for systematic analysis, on the other hand, they do not allow one to assess temperature-dependent relaxation processes or to obtain activation parameters by the method of isolation. The research will be extended to a broader temperature and compositional range to achieve a thorough understanding of molecular dynamics, and such an experiment is part of our future work plan. It should be noted that the present work focuses on dielectric characterization, and direct evaluation of pharmaceutical performance parameters such as dissolution, permeability, or bioavailability was not undertaken. The discussion is focused on fundamental physicochemical insights into solvation behaviour and molecular mobility as reflected in the dielectric response of the system. While these findings may have relevance for pharmaceutical environments, particularly in understanding solute-solvent interactions, any implications for drug delivery or formulation performance are presented cautiously, as such applications require direct experimental validation beyond the scope of the present study.

## 4 Conclusion

The present investigation offers a detailed dielectric measurement for various mixtures of APAP-DMSO from frequency 10 Hz-10 MHz at temperature 25 °C. Obtained data was analysed and interpreted based on the characteristics of solute-solvent interaction. A regular behaviour changing with change in molarity of APAP has been observed by all samples for the dielectric measurements. Electrode polarization phenomena are observed for different molarity solutions in complex permittivity and loss tangent spectra across the frequency domain. Analysis of the complex permittivity response enabled identification of electrode and conduction polarization processes, along with the calculation of their relaxation times Cole-Cole fitting is also done to determine the fitting parameters and relaxation time from the real permittivity curve. The impedance analysis clearly demonstrates that molar concentration plays a critical role in governing interfacial charge transport. A systematic increase in charge-transfer resistance (Rct) with decreasing concentration indicates progressively hindered charge-transfer kinetics and reduced charge carrier mobility. Simultaneously, the pronounced rise in double-layer capacitance (Cdl) at lower concentrations reflects enhanced interfacial charge accumulation, increased electrode polarization, and a higher density of trapped charges at the interface.

The concurrent increase in Rct and Cdl signifies that although charge carriers accumulate at the interface, their ability to cross the interfacial barrier is severely restricted, leading to mobility-limited transport. The emergence of non-ideal interfacial behaviour at lower concentrations, as evidenced by the requirement of a constant phase element, further confirms increased heterogeneity and dispersive charge dynamics. The results establish a clear correlation between concentration-dependent interfacial resistance, polarization effects, and charge carrier mobility, highlighting the dominant role of interfacial processes in determining the electrical transport properties of the system. These physicochemical properties are qualitatively consistent with processes such as dissolution, molecular dispersion, and diffusional dynamics within the system. These observations may provide useful insight into solvent–solute interactions relevant to formulation design, although direct implications require further validation.

## Data Availability

The raw data supporting the conclusions of this article will be made available by the authors, without undue reservation.
