# Supplementary figures and images for: Physicochemical behaviour of acetaminophen-Dimethyl Sulphoxide mixtures studied via dielectric spectroscopy
Source: Front Chem. 2026 May 29;14:1733069. doi: 10.3389/fchem.2026.1733069 (PMC13261813; doi:10.3389/fchem.2026.1733069)

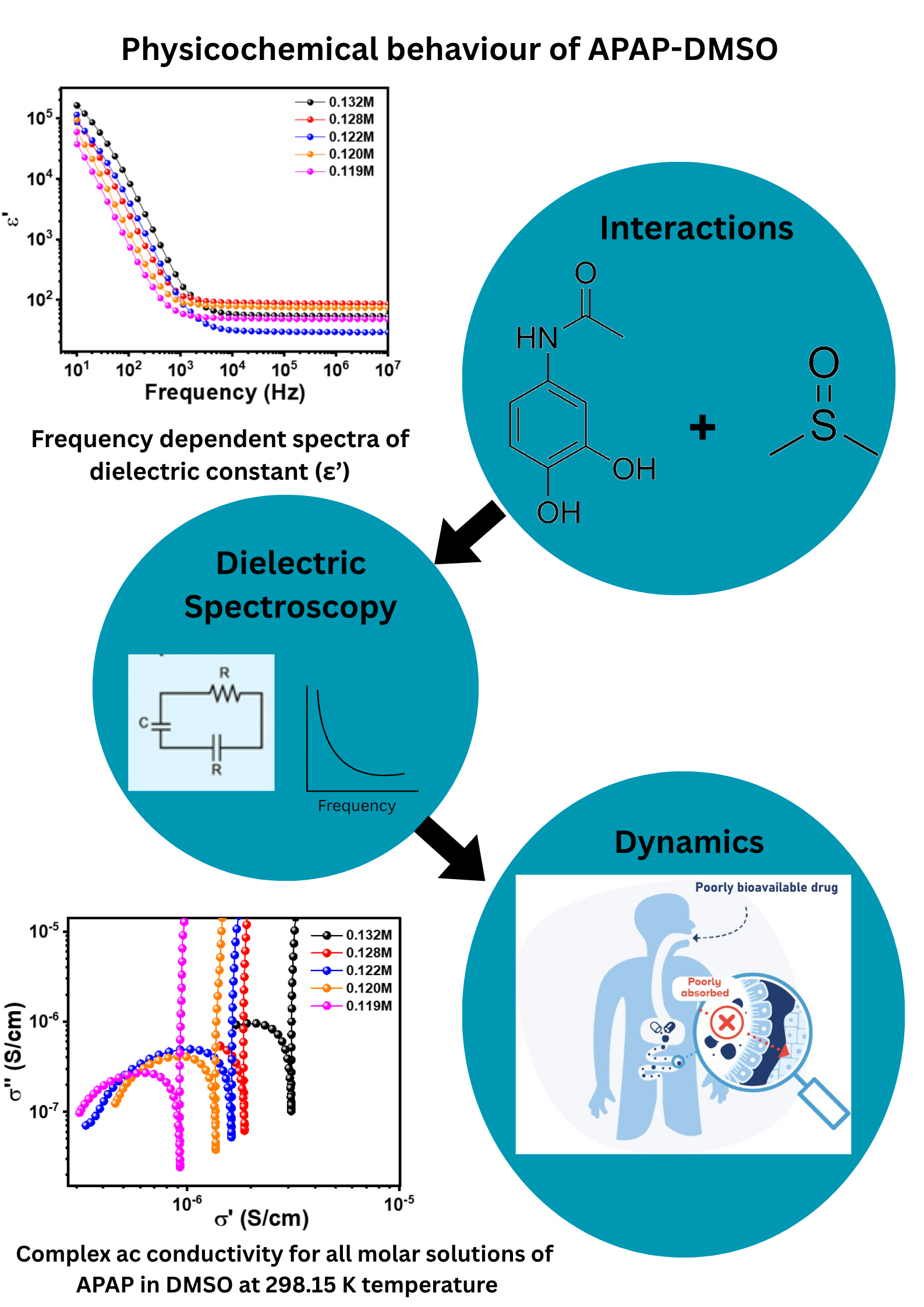

Supplement: Supplementary file 1 [file Supplementaryfile1.png]
